# Supplementary figures and images for: Metabolic and Oxidative Stress Management Heterogeneity in a Panel of Breast Cancer Cell Lines
Source: Metabolites. 2024 Aug 6;14(8):435. doi: 10.3390/metabo14080435 (PMC11356218; doi:10.3390/metabo14080435)

**FIGURE S1**

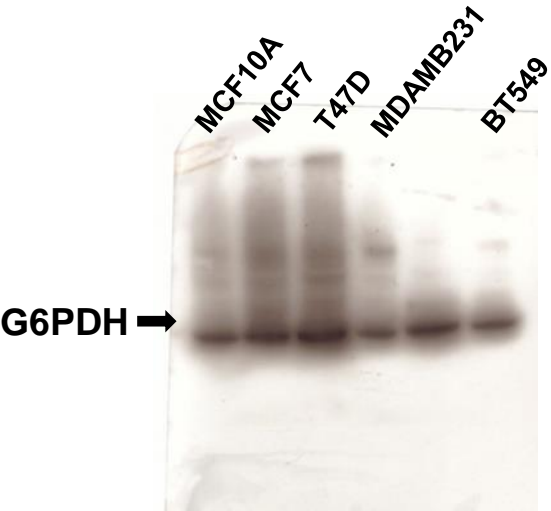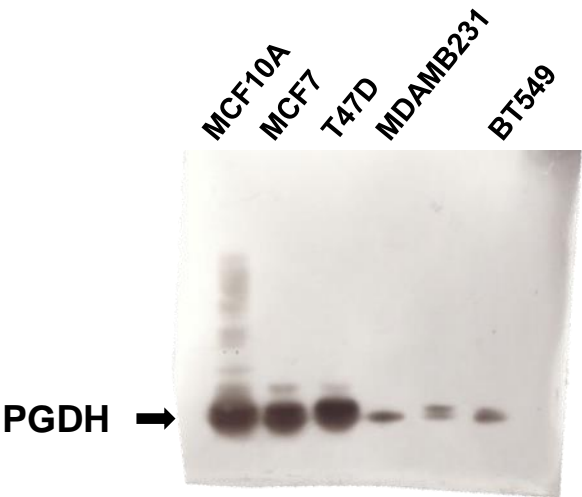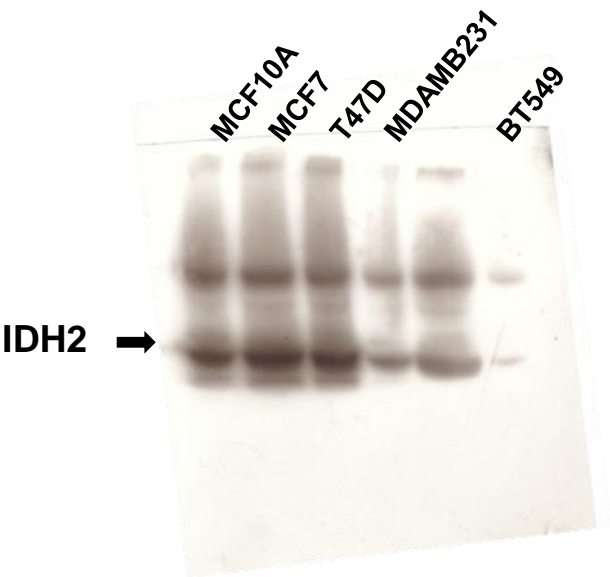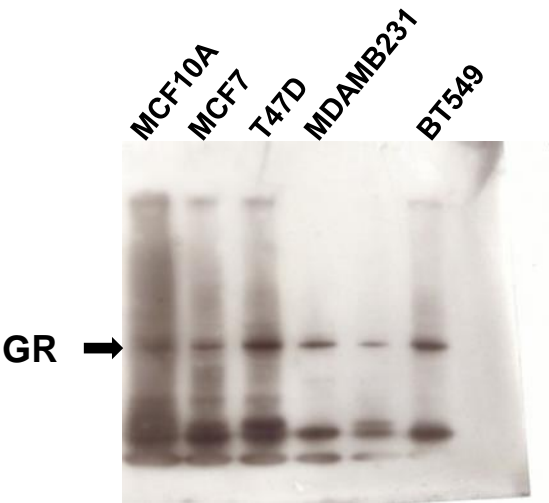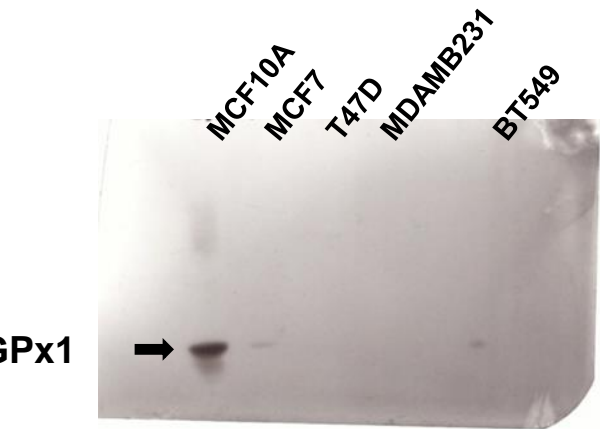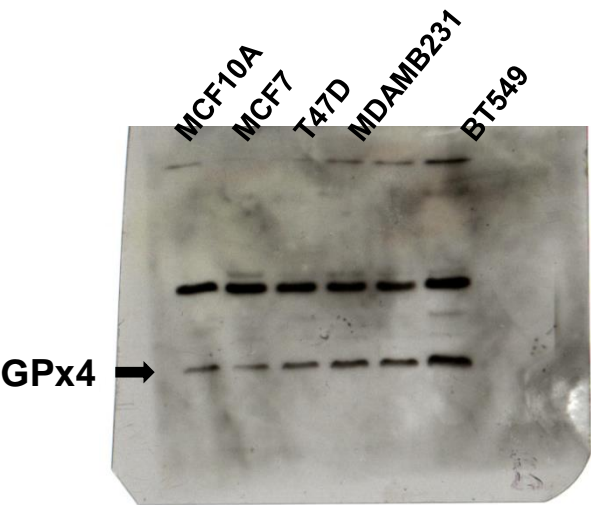

SOD 1

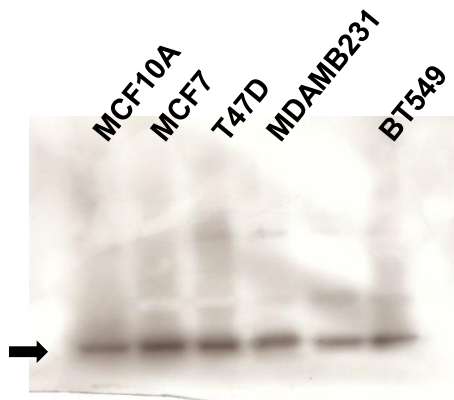

SOD 2

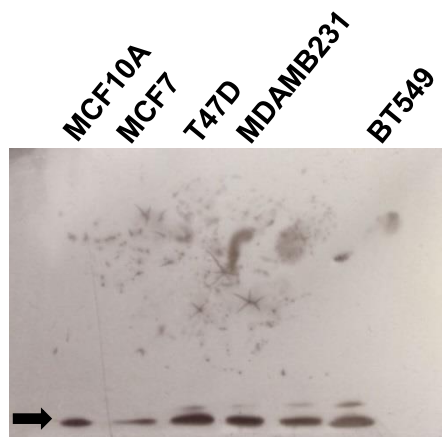

CAT

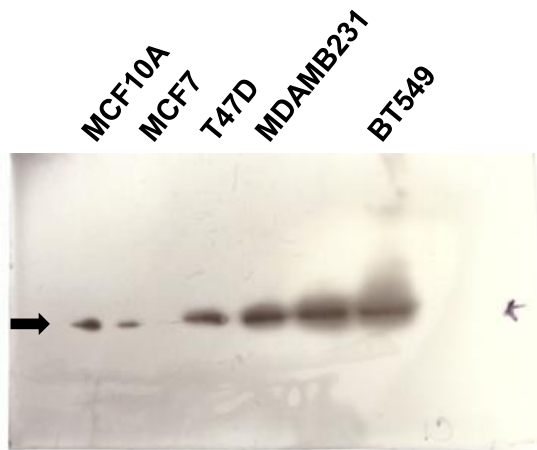

HKII

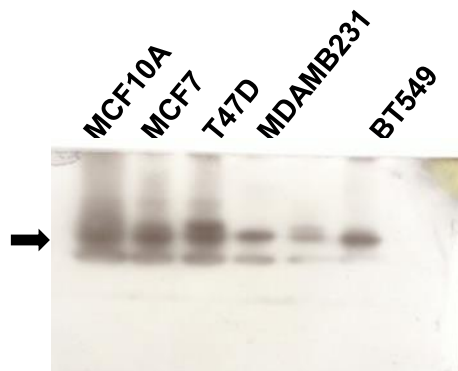

CS

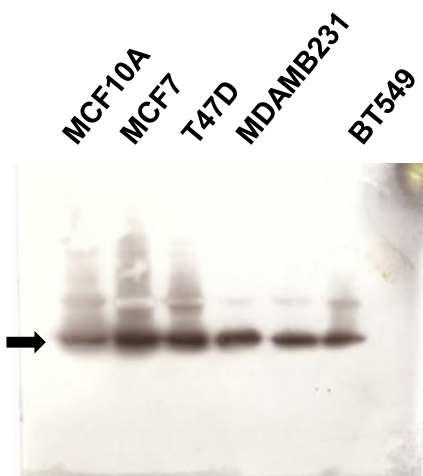

$\beta$ -actin

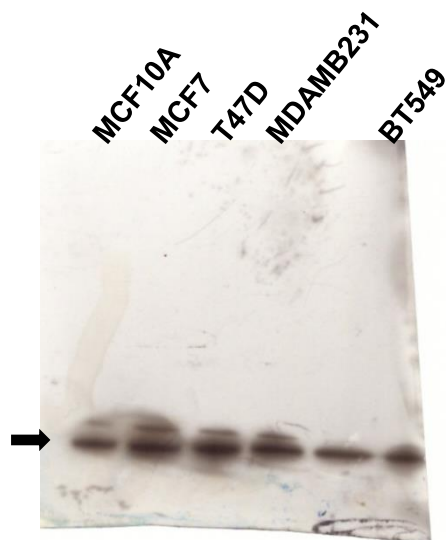

Supplement: Supplementary file 1 [file metabolites-14-00435-s001.zip › metabolites-3115684-supplementary.pdf]
